# STEM approach using soccer: improving academic performance in Physics and Mathematics in a real-world context

**DOI:** 10.3389/fpsyg.2025.1503397

**Published:** 2025-02-24

**Authors:** Miguel Ángel Queiruga-Dios, José Benito Vázquez Dorrío, María Consuelo Sáiz-Manzanares, Emilia López-Iñesta, María Diez-Ojeda

**Affiliations:** ^1^Department of Specific Didactics, University of Burgos, Burgos, Spain; ^2^Department of Applied Physics/IFCAE, University of Vigo, Vigo, Spain; ^3^Department of Health Sciences, University of Burgos, Burgos, Spain; ^4^Department of Didactics of Mathematics, University of València, Valencia, Spain

**Keywords:** gender, Mathematics, Physics, STEM, real-world contexts, secondary education, academic performance

## Abstract

This proposal adds original approaches to the currently scarce body of practical evidence on the application of STEM innovations in the curriculum. A teaching-learning program was designed in a real-world context such as the game of soccer with a STEM (Science, Technology, Engineering and Mathematics) approach through a cooperative problem-solving methodology. The objectives of the research focus on analyzing the effect of the use of this STEM unit on the academic performance of students, taking into account the gender variable; and their appreciation of the activities and methodology used, as well as the challenges encountered and their solutions. The intervention was implemented in the 4th year of Compulsory Secondary Education in a school in Spain with 36 students (24 girls and 12 boys). Academic performance was analyzed taking into account the gender variable, for which a quasi-experimental design was applied before and after with a control group. The appreciation and interest of the experimental group regarding the methodology used as well as the difficulties that arose were studied. As a result, there is an improvement in the academic performance, which is more evident in girls. The methodology has been valued positively and the greatest difficulties refer to the distribution of roles and understanding and carrying out the activities, however, these difficulties were resolved with the help of classmates and the teacher.

## 1 Introduction

In Spain, Physics and Mathematics are among the scientific subjects in Compulsory Secondary Education (CSE) that exhibit the lowest academic performance index [Ministerio de Educación y Formación Profesional (MEFP), 2020]. The difficulties are apparently explained by the abstraction of the scientific contents involved and the previous acquisition by the students of erroneous concepts, which inhibit the correct construction of new knowledge (Suprapto, 2020). However, the use of digital and technological tools has the potential to improve teaching-learning procedures in these disciplines by promoting the construction of new concepts (Bicer and Lee, 2023; Queiruga-Dios et al., 2021a, 2021b). In order to produce meaningful learning (Ausubel et al., 1976/1983), students must see the information they receive as relevant to their daily lives in such a way that the modification of previous knowledge leads to new learning (Gravemeijer et al., 2017). Problem-solving contributes to a conceptual change in learners, favored by the cognitive conflict it provokes (Jonassen et al., 2005). Cooperative learning, like “the instructional use of small groups so that students work together to maximize their own and each other’s learning” (Johnson and Johnson, 1999, p. 72), also plays an important role in meaningful learning, producing a favorable attitude toward learning in the pupils and increasing their performance among both the more gifted and those with difficulties (Bicer and Lee, 2023; Radulović et al., 2022). Thus, in this socialization context, the basic elements of cooperation are (Johnson and Johnson, 1999): (1) Positive interdependence among the team members, so that the achievements are obtained with the participation of all of them; (2) Individual accountability, each student is individually evaluated; (3) Face-to-face promotive interaction, so that students in each group support each other; (4) Social skills, that allow the success of learning in cooperative work; and (5) Group processing, through discussions and reflections among group members on what actions are most appropriate to obtain achievements. Likewise, Physics and Mathematics can be integrated with educational technologies and a suitable methodology in a STEM (Science, Technology, Engineering, and Mathematics) approach in a way, that is comprehensive and connected through real-world topics and real-world connections to what students are learning and in this way the links between scientific content and everyday life can be appreciated by students (Dare et al., 2021; King and Ritchie, 2012; Roehrig et al., 2021). The importance of such integration of disciplines by using real-world contexts is reflected in the PISA assessments. In Mathematics, the test focuses on measuring the capacity of students to use diverse contexts to describe, explain, and predict phenomena (OECD, 2018, 2019, 2023). The performance measured here is related to the students’ ability to extrapolate from their knowledge in different situations, thus involving more than just the ability to reproduce concepts and procedures acquired in the classroom. Thus, most Mathematics units in PISA refer to real-world contexts in which mathematical skills are needed to solve a specific problem, using the technological tools that are also available in the real-world (e.g., calculators, rulers, or spreadsheets). Likewise, Science units in PISA attempt to measure the students’ capacity to participate in matters relating to science. Consequently, according to Spanish legislation [Ministerio de Educación y Formación Profesional (MEFP), 2020; OECD, 2019] students are now required to have knowledge of standard scientific methodological procedures for problem-solving, such as comparatively assessing or research design, and scientific interpretation of the results obtained from experimentation.

There is a lack of curricular incorporation of integrated STEM approaches in secondary education, since it is necessary, at least, an additional investment of time and effort in integration, the use of new instructional practices and collaboration between subjects, which requires teacher training (Rennie et al., 2018; Shernoff et al., 2017; Thibaut et al., 2018). In this study, however, a way is presented for the application of an integrated STEM approach that does not require a large deployment of interdisciplinary or technological means, instruments and knowledge, but rather, through a simple and real-world context, such as soccer, and using everyday tools, it generates a connection between STEM disciplines.

This article analyses the performance of pupils in 4th-grade Physics and Mathematics in CSE, considering the gender variable, after implementing a Teaching Unit, with Physics contents related with kinematics, force and energy, designed following a constructivist model based on the following five axes: (1) digital and technological tools, (2) make sense of the information received through connection with real situations, (3) problem solving, (4) cooperative learning, and (5) STEM integration. The scientific literature indicates that when educational problem-solving activities using technologies are designed, the learners’ performance improves (Bicer and Lee, 2023; Hochberg et al., 2018; Queiruga-Dios et al., 2021b) and so the research will be broadened to incorporate a real-world context that is an everyday reality for the student. Thus, in activities designed in an integrated way, learners face everyday problem situations as if they were a scientific team that has solved them cooperatively with the help of digital and technological tools. These activities were created with the well-known game of soccer as the real-world context in which the contents of the subjects of Physics and Chemistry, and Mathematics (MECD, 2015, 2022), among others, are developed. At the same time, considering that soccer is a sport that is often related to a male stereotype in many countries, gender variable is also taken into consideration. Finally, the assessment and interest of the students in the proposed activities and the approach used are analysed, as well as the challenges that arose and how they were solved.

## 2 Theoretical framework

The term integrative STEM education refers to the teaching of Science and Technology disciplines in an integrated and connected way in the context of technological designing/problem solving (Sanders, 2009, 2012). STEM education allows students to learn conceptual syllabus materials as they develop 21st-century skills such as communication, critical thinking, creativity, and collaboration (Gravemeijer et al., 2017; MacDonald et al., 2019). Then, it is presumed, that they will be able to join the social and employment world efficiently, prepared to solve problems in a changing global environment. This requires a non-fragmented knowledge of the disciplines that allows for the transfer of knowledge between subjects, enabling learners to understand how they are connected (Bybee, 2010; Radulović et al., 2022).

There are calls from various quarters for an integrative approach to STEM education that is applicable to the real-world to solve the global challenges and problems of the modern world (Bybee, 2010; Gravemeijer et al., 2017; Radulović et al., 2022). In this sense, it is often pointed out that science is fundamentally oriented toward solving problems and challenges (Morrison et al., 2020; Vorhölter and Krüger, 2021). Therefore, the way that scientific teams face real-world problem situations with the same processes and strategies used in the lab can be included in the study of science in the classroom. The creation of a space in schools for research-based methods will have a positive effect on the decline in STEM vocations that society has been undergoing for several years (Bicer and Lee, 2023; Rocard et al., 2007). In the case of Physics, the gender gap existing in scientific-technological studies is more marked than in other disciplines at both the university and pre-university levels. That gap is reduced, while the performance of both girls and boys is enhanced, by using interactive teaching-learning methods that promote collaboration and place emphasis on conceptual understanding (Radulović et al., 2022).

Putting into practice how to approach everyday problem-solving, while at the same time allowing the acquisition of mathematical knowledge stimulates the development of logical-mathematical thinking in pupils (Gravemeijer et al., 2017). It also favors a significant conceptual change in the students by carrying out the stages of analysis of the problem, hypothesis development, and analysis of the results. All this with metacognition strategies brought into play through verbalisation of processes, feedback, and discussion (Sáiz-Manzanares et al., 2019; Vorhölter and Krüger, 2021). It is therefore necessary that when they design activities, teachers take into account the importance of all those stages, creating spaces and times for the pupils to explain and justify the processes followed to resolve the problems and analyse the solutions. Moreover, this should be done in such a way that the pupils understand what procedures have, or have not, led them to the correct answer. It can be said, regarding academic performance, that the didactic methodology used is a determining factor in the implementation of problem-solving in the classroom. The learners’ attitude will determine how their problem-solving abilities develop as a result of this (Gravemeijer et al., 2017).

At the same time, cooperative work is a vital strategy for a constructivist approach to learning. The use of cooperative learning in the field of experimental sciences offers advantages in terms of learner motivation and improved academic results. A fundamental process of this learning is the collaborative co-construction of ideas and meanings among group members. In this way, the students jointly produce new ideas by reviewing, discussing, and improving each other’s contributions. In this way, group performance and learning are greater than an individual student would achieve alone, so an individual’s learning and performance are improved. In this working context, ICT tools favor the exchange and analysis of information while improving students’ attitudes toward science (Bicer and Lee, 2023; Radulović et al., 2022).

### 2.1 STEM from a gender perspective

The UNESCO report *Cracking the code: Girls’ and women’s education in Science, Technology, Engineering and Mathematics (STEM)* states that girls (72%) and boys (75%) are equally committed to STEM disciplines in the 10-11-year old age. However, at the age of 18 these percentages decrease and differ dramatically (19% for girls and 33% for boys). As a result, women’s interest in STEM disciplines has declined. Subsequently, at university, the difference between enrolments in, for example, Engineering, Technology, Construction and Computer Science decreases for women and increases for men (28% vs. 72%) (Bokova, 2018). Several studies on the gender gap existing in STEM disciplines (Barone and Assirelli, 2020; Redmond and Gutke, 2020; Weeden et al., 2020) show a series of factors that favor this situation. One of the most common factors is the difference in academic performance between women and men in Science and Mathematics disciplines during the years leading up to university. In this case, the theory of rational choice (Stearns et al., 2020), which suggest that people tend to favor educational choices that boost their prospects of success, would justify women orienting their degree choice toward the arts and humanities, where they gain better grades. Additionally, the better grades obtained by women in social sciences would also represent a competitive advantage for them over men, which would lead to them selecting degrees in those fields rather than STEM (Vaarmets, 2018). However, this hypothesis is not conclusive and requires greater research (Barone et al., 2017). It should be noted that the differences in academic performance in STEM disciplines when considering the gender variable are not large enough to justify the gap that exist and explain it in terms of the male advantage in Mathematics and Science (Thébaud and Charles, 2018; Weeden et al., 2020). Thus, other authors place greater relevance on the self-assessment done by the individuals themselves regarding mathematical and scientific skills (Weeden et al., 2020). In this sense, women self-assess their mathematical ability less positively than men do; regardless of the real grades they obtain (Thébaud and Charles, 2018). Positive self-assessment increases self-efficacy and maintains motivation. The notion of self-efficacy has been employed to propose that students who possess the belief that they can succeed will opt for science subjects and study them, even later on in their careers. Scientific success and perseverance in the field can be predicted by self-efficacy (Redmond and Gutke, 2020). This occurs in the years leading up to university entrance and causes boys to develop a higher level of self-confidence and self-efficacy in Mathematics (Eccles, 2011), which would justify their greater inclination toward STEM degrees, whereas girls who think they are bad at Mathematics will not aspire to take degrees in that subject or the sciences.

Other factors in the gender gap are related to girls’ beliefs that they will face greater discrimination in STEM degrees, traditionally pursued by men. In this sense, the influence of significant references (teachers, parents, and advisors) plays an important role, particularly toward the beginning of a student’s schooling, when they are the youngest (Barone and Assirelli, 2020; Weeden et al., 2020). These gender differences in treatment may occur in terms of monitoring, mentoring, or support for research activities. In these activities, men seem to benefit, which perpetuates male dominance in STEM (O’Connor et al., 2020). In short, there is a climate that favors and fosters boys rather than girls (Clark-Blickenstaff, 2005).

On the other hand, women in countries with greater empowerment are less inclined to make a choice related with Mathematics and Science professions (Chang and Chang Tzeng, 2018). This is related to the different interests expressed by gender for the different topics or contexts in which the different disciplines are approached (Lavonen et al., 2010). This should be taken into account to create female-friendly disciplines (Naukkarinen and Bairoh, 2020), while the syllabus materials used in science education should be properly analysed and prepared to avoid gender bias and improve, in general, the educational pedagogy of STEM disciplines (Clark-Blickenstaff, 2005). In addition, studies on integrative STEM experiences show an increase in the achievement of all students of different characteristics in Mathematics and Science (Becker and Park, 2011).

Considering the above, it is hypothesized for this research that the implementation of integrated activities related to the well-known game of soccer, as a real-world context, and connected to the subjects of Physics and Mathematics will lead to an increase in student performance, especially girls’ performance, in these subjects. Moreover, this will be accompanied by an improvement in interest in the disciplines and an appreciation for the methodological approach used.

## 3 Research objectives

The objectives proposed for this research were as follows:

- Investigate the effect of using the STEM unit created about the sport of soccer by employing cooperative learning on students’ academic performance, taking the gender variable into consideration.
- Investigate the effect of the same unit on students’ appreciation and interest regarding the proposed activities and the methodology used, as well as the problems encountered and their solutions.

## 4 Methodology

This section describes the participants in the research, the instruments used, and the data analysis carried out. The students’ usual way of solving problems is cooperatively, for which they have the necessary tools, such as computers with internet access and office software. The difference between the control group and the experimental group is that the real soccer context was incorporated in the latter. This will allow us to answer the research question regarding how the soccer context and organizing the content under a STEM approach influences the academic performance of the students and what the students’ appreciation is.

### 4.1 Participants

A before-and-after quasi-experimental design with a control group was applied. The experimental group is made up of 36 individuals (24 girls and 12 boys), and the control group is made up of 35 individuals (23 girls and 12 boys), all students in the 4^th^ grade of CSE (15–16 years). Pupils were assigned to the sample by convenience sampling, as the students remained in their corresponding groups. While the control group, worked with the Physics and Mathematics syllabus by resolving cooperative problems and using Technology (Queiruga-Dios et al., 2021b), the experimental group held class sessions with the same approach but contextualized within the game of soccer as a real-world context. The main difference in the work between the two groups was that the control group worked on a variety of problems without a defined context, while in the experimental group all activities were linked to the real context of the ball game and to situations that are usually found in soccer. In both cases, the didactic proposals were used exclusively to teach the contents, without the complementary use of other more traditional tools as it could be exclusively the resolution of exercises within the classroom.

In the design of the Unit for the experimental group, the disciplines appear integrated under the integrative STEM approach (Sanders, 2009, 2012). The summary of the main contents studied is provided as complementary material (Supplementary material 1). These contents have been developed in such a way that the student can connect them with the prior knowledge of Physics and Mathematics (employed not only as a useful tool but also used conceptually) acquired in previous courses. Content management requires that involved students design and analyse their own experiments during the development of cooperative tasks (Bybee, 2010), using technological tools (Vieyra Software, 2020; Tracker, 2020) and with an engineering perspective, where the problems are solved by processes that provide innovative solutions, including the construction of instruments and the performance of necessary tests, as defined by the National Academy of Engineering and National Research Council (NRC) (2014). To carry out these activities, the pupils formed groups of four and assumed the following roles in each team: coordinator, secretary, reporter, and material supervisor. The roles were rotated and changed for each practical session. In that way, each pupil had to perform all the tasks.

The implementation in the classroom of the STEM Unit for the experimental group as well as the development of the class sessions can be found in the Supplementary material 2. The design of the STEM Unit for the experimental group provides the integration of Mathematics and Physics, depending on the activities, at a multidisciplinary, interdisciplinary, or transdisciplinary level. However, Technology and Engineering appears at a level of transdisciplinary integration (Supplementary material 2).

### 4.2 Instruments

Consent was obtained from the Management of the School. Likewise, all the pupils and their families were informed of the objectives of the study, and their consent was acquired, which also covered the use of photos to document the experiments.

To develop the research and data collection, the following instruments were used:

- Teaching Unit for the syllabus for Physics and Mathematics contextualized to the subject of soccer. As indicated, this was designed with a constructivist approach so that the pupils had to carry out a series of experiments or solve problems that permitted distinct approaches and different solutions (Queiruga-Dios et al., 2018).
- Questionnaire on the syllabus in order to measure the pupils’ academic performance. It is provided as Supplementary material 3. Comprising eight questions on the Physics and Mathematics syllabus, it measured the degree of conceptual comprehension of the pupils. The questionnaire was designed on the basis of known tools that measure whether the pupils master a specific set of concepts: *Test of Understanding Graphs in Kinematics for High School* (TUG-K2) (Beichner, 1994), *Force Concept Inventory* (FCI) (Halloun et al., 1995), *Force and Motion Conceptual Evaluation* (FMCE) (Thornton and Sokoloff, 1998), and *Energy Concept Assessment* (ECA) (Ding et al., 2013). It was adapted to the age group of the pupils and later reviewed and validated by six teaching staff members, specialists in teaching secondary school Physics. The correspondence between the questions on this questionnaire and previous ones is shown in Table 1.
- Questionnaire on students’ appreciation of the activity and the methodology used. This questionnaire comprised 9 multiple-choice questions (1 to 9), which used a Likert-type scale with values ranging from 1 – *totally disagree* to 4 – *totally agree*, and four open questions (10, 11, 12 and 13).

**Table 1:** Correspondence between the questions created for this experiment and the original ones.

Question	Original test question
1	1 FCI
2	19–20 of FCI; 27–29 FMCE
3	2 TUG-K2
4	17 TUG-K2
5	1 ECA
6	8 TUG-K2
7	2 ECA
8	7 TUG-K2

### 4.3 Data analysis

This research is quasi-experimental, with a quantitative-qualitative approach and with a control group and an experimental group. Data analysis was conducted using the SPSS v.24 statistics package for questions 1 to 9. The Alpha coefficient was 0.7325, a value that indicates acceptable internal consistency for the initial stages of research or exploratory studies (Nunnally, 1967). For the quantification 10 to 13, the categorization was carried out after analysis of the answers. For this, two experts read the students’ answers and established the categories presented in Table 2. A procedure based on grouded theory (Trinidad et al., 2006) was used to categorize the students’ responses. In this way, the students’ responses were collected, allowing for an initial classification according to similarity. Using this first categorization, a constant comparative method was used, establishing response–response, response-category, and category-category comparisons until the responses did not add information to the categories generated, formulating a definition of each category. Finally, two independent judges are provided with the definitions of the categories and the literal transcription of the statements made for their classification.

**Table 2:** Categorization of the answers to questions 10–13 of the questionnaire on appreciation and interest.

Question	Categories	Explanation
A1. What difficulties have you had carrying out the activities?	A1.1. Technical difficulties.	The student had difficulties with the use of Technology to perform tasks or take measurements.
	A1.2. Difficulties with the activities (comprehension).	Difficulties are related to the way of approaching the situation posed in the task.
	A1.3. None.	The student had no difficulty.
	A1.4. Organizational in terms of fulfilling the roles.	The student had difficulties in organizing himself/herself according to the assigned roles or small conflicts within the group.
	A1.5. Lack of time.	The student would have preferred more time for homework.
A2. How did you overcome the difficulties that arose?	A2.1. Were not overcome.	The student did not manage to overcome the difficulties encountered.
	A2.2. Were overcome with the help of teammates.	The student asked his/her classmates for help.
	A2.3. Were overcome by dialog.	The student solved the difficulties by talking.
	A2.4. Internet search.	The student solved the difficulties by searching for information and documentation on the Internet.
	A2.5. Asking the teacher.	The student asked the teacher for advice.
A3. Thinking about what you have learned. Will you be able to apply it in your life in the future? If you answer ‘yes’, then how?	A3.1. Do not know.	The student replied “I do not know”.
	A3.2. He/she will be able to apply it in his/her daily life.	The student believes that he/she will be able to apply what he/she has learned to his/her daily life.
	A3.3. He/she will be able to apply it in their academic life.	The student considers that what he/she has learned will be useful in his/her academic life.
A4. Final reflections and suggestions.	A4.1. The activity has been very appropriate and he/she has enjoyed it a lot.	The student has enjoyed the activities and homework.
	A4.2. Team organization could be better	The student states that the organization of his/her team could have been more appropriate.
	A4.3. Include more feedback sessions.	The student indicates that he/she would have liked more brainstorming and review sessions.

Because the sample is less than 50 individuals in each group, the Shapiro–Wilk test was applied to determine if the data shows a normal distribution (de Souza et al., 2023). The Shapiro–Wilk test indicated that the difference between the data sample and the normal distribution is not statistically significant for both the control group (W (35) = 0.97, *p* = 0.474, Skewness = 0.49; Excess kurtosis = −0.16) as for the experimental group (W (36) = 0.95, *p* = 0.129; Skewness = −0.86; Excess kurtosis = 2.43). Levene’s test confirmed that the experimental group and the control group are equivalent in the pretest condition (*F* = 1.12, *p* = 0.33), so the requirement of homogeneity is met.

## 5 Results

Below are descriptions of the results corresponding to each objective:

*Objective 1: Investigate the effect of using the STEM unit created about the sport of soccer by employing cooperative learning on students’ academic performance, taking the gender* var*iable into consideration.*

With respect to the experimental group, a comparison of the pre-and post-test results enables the analysis of the acquisition of competences using the STEM unit on the Physics and Mathematics syllabus. Table 3 shows the data for the experimental group.

**Table 3:** Results of the pre-test and post-test on the Physics and Mathematics syllabus in the experimental group.

		Pre-test		Post-test		Difference		*t*-student	*p*	d Cohen
	*n*	*M*	SD	*M*	SD	*M*	SD			
Total	36	2.61	1.10	5.42	1.80	2.81	1.74	9.69	0.00001*	1.88
Girls	24	2.29	0.91	5.38	1.58	3.08	1.38	10.94	0.00001*	2.39
Boys	12	3.25	1.22	5.5	2.26	2.25	2.26	3.45	0.00546	1.24

^*^p < 0.001.

M = mean, SD = standard deviation. Interpretation: *d* = 0.2–0.3, small effect value; *d* = 0.5 medium effect value; and d ≥ 0.8, large effect value.

It is evident that the starting situations based on gender in the pre-test exhibit a significant disparity of almost one point (0.96) in favor of the boys. However, significant differences for the girls can be detected in the post-test results (*p* = 0.0001 to a confidence level of 99.9%) but not for the boys. Likewise, the effect value is high (Cohen, 1992) for both girls and boys. Table 4 shows the data for the control group.

**Table 4:** Results of the pre-test and post-test on the Physics and Mathematics syllabus in the control group.

		Pre-test		Post-test		Difference		*t*-student	*p*	d Cohen
	*n*	*M*	SD	*M*	SD	*M*	SD			
Total	35	3.94	1.54	5.09	1.55	1.14	1.29	5.24	0.00001*	0.74
Girls	23	3.7	1.63	4.84	1.41	1.16	1.17	4.78	0.00004*	0.75
Boys	12	4.47	1.27	5.57	1.75	1.1	1.56	2.45	0.01611	0.72

^*^p < 0.01.

M = mean, SD = standard deviation. Interpretation: *d* = 0.2–0.3, small effect value; *d* = 0.5 medium effect value; and d ≥ 0.8, large effect value.

Also in this case, the starting situation in the pretest differs by 0.77 points in favor of boys, and we see significant differences in girls’ performance, although smaller than in the previous case. In this case, the effect size is moderate, smaller than for the experimental group.

To complete the above-mentioned outcomes, the responses to the questionnaires given to pupils were examined. In order to determine whether pupil performance had improved, worsened, or remained the same after the educational intervention, a new classification for pupil performance was established based on the number of correct or incorrect answers in the pre-test and post-test: 86, 8 and 6%, respectively. If gender variable is considered a large improvement in the academic performance of the girls (95.8%) compared to the boys (66.7%) is observed.

An ANCOVA was performed to examine the effectiveness of the intervention while controlling for pre-test scores in both the experimental and control groups. The mean score for the pre-test was higher in the control group (*M* = 3.94; SD = 1.54) than in the intervention group (*M* = 2.61; SD = 1.10). However, following the STEM Unit, the experimental group outperformed their counterparts in the control group (*p* = 0.004), with a medium to large effect size as indicated by the partial eta squared of 0.118. The adjusted mean scores for the post-test were 5.83 (SE = 0.26) for the experimental group and 4.47 (SE = 0.26) for the control group. Overall, it can be concluded that the intervention was effective in improving students’ performance. These results suggest that, before the intervention, the control group had a higher score. After the intervention, the opposite was true. The experimental group improved their score and the control group did not. And then it is the experimental group that has a higher score.

In both groups, normalized gain was calculated (Hake, 1998) from the mathematical expression 𝑔= (*𝑝𝑜𝑠t-𝑡est* − *𝑝𝑟𝑒-test*)/(*maximum score* − *𝑝𝑟𝑒test*), where *post-test* and *pre-test* are the average scores obtained by the pupils in the pre-and post-tests respectively, and *maximum score* is the maximum score that can be obtained in the tests. Table 5 shows the results obtained.

**Table 5:** Normalised gain values for the whole group and by gender in experimental and in control groups.

Experimental group			Control group		
Total gain	Gain girls	Gain boys	Total gain	Gain girls	Gain boys
0.52	0.54	0.47	0.30	0.30	0.33

Low normalised gain (g < 0.3), medium (0.3≤𝑔<0.7) and high (g ≥ 0.7).

While the gains for boy and girls in the control group are similar, it can be seen that the gain for girls in the experimental group is greater (0.54) compared to that for boys (0.47). Table 6 shows the distribution of these gains according to Hake’s categorization.

**Table 6:** Distribution of normalised gain according to ranges in Hake’s categorization in experimental and in control groups.

	Experimental group			Control group		
Gain range	Total	Girls	Boys	Total	Girls	Boys
g < 0.3	16.7%	8.3%	33.3%	31.4%	47.8%	41.7%
0.3 ≤𝑔<0.7	58.3%	70.8%	33.3%	45.7%	47.8%	41.7%
g ≥ 0.7	25%	20.8%	33.3%	8.6%	4.4%	16.7%

In the control group, it can be seen that both for girls and boys the greatest percentages of gains are equally distributed among the low and medium gain ranges. At the same time, in the experimental group, while most girls are in the medium gain range (70.8%), the gains for boys are uniformly spread over the three intervals. The findings of the experimental group’s students’ pre-and post-tests will next be carefully examined.

**Figure 1:**
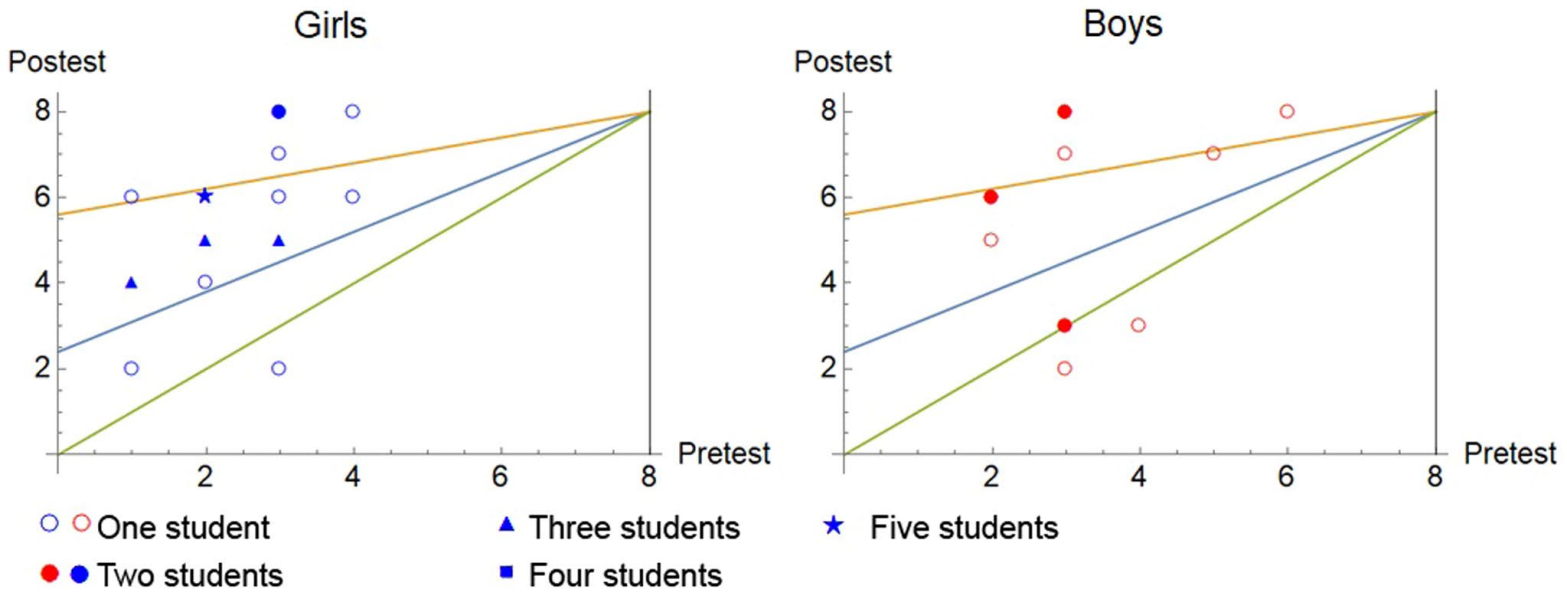
Representation post-test against pre-test considering the gender variable in experimental group.

Figure 1 shows the post-test score versus the pre-test score, separated by gender and grouped by value. The straight lines, from bottom to top, represent specific gains respectively, zero, 0.3 and 0.7. The figure shows how, despite having more boys with the maximum scores in the post-test, girls are positioned in a larger proportion in the medium gain area.

**Figure 2:**
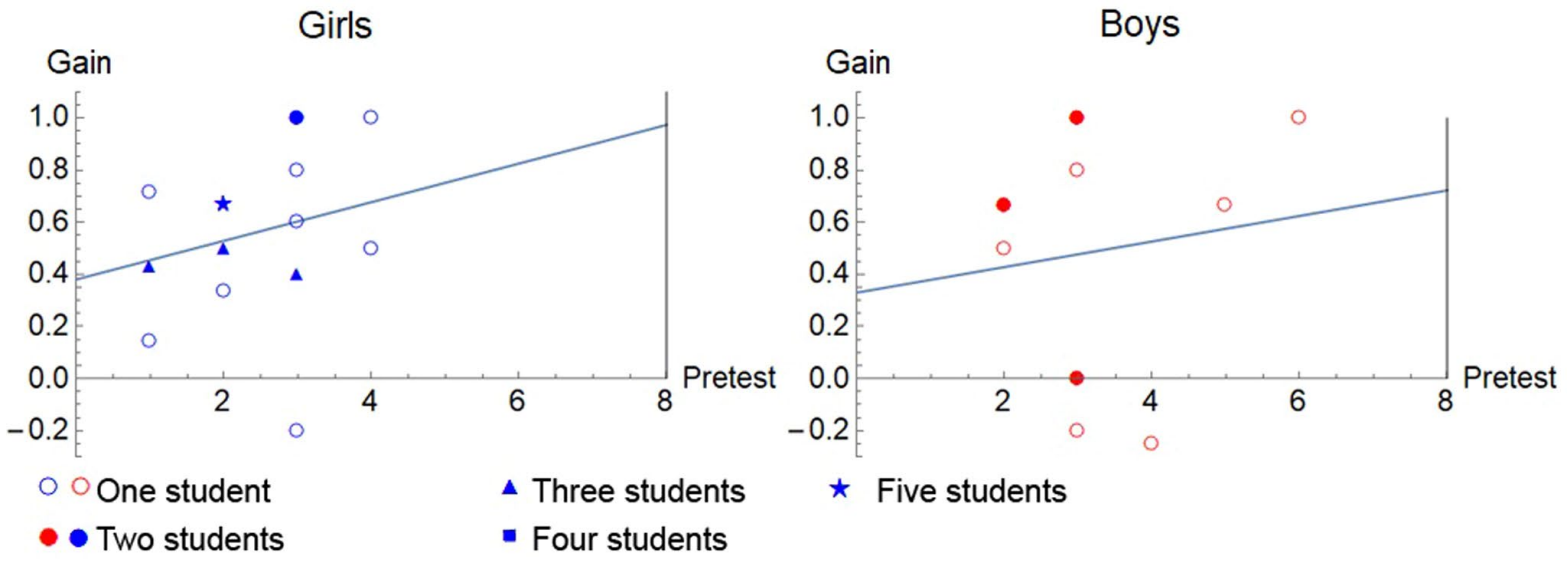
Representation of the gain against the pre-test scores and their corresponding regression line in experimental group.

A similar comparison, considering the gender variable, between the gain and the results of the pre-test and post-test shows the dispersion of the sample. Figure 2 shows a high dispersion for both genders together with a weak relationship between the gain and the results, being higher for the girls in this case, with a correlation coefficient for girls of *r* = 0.25 and for boys of *r* = 0.12.

**Figure 3:**
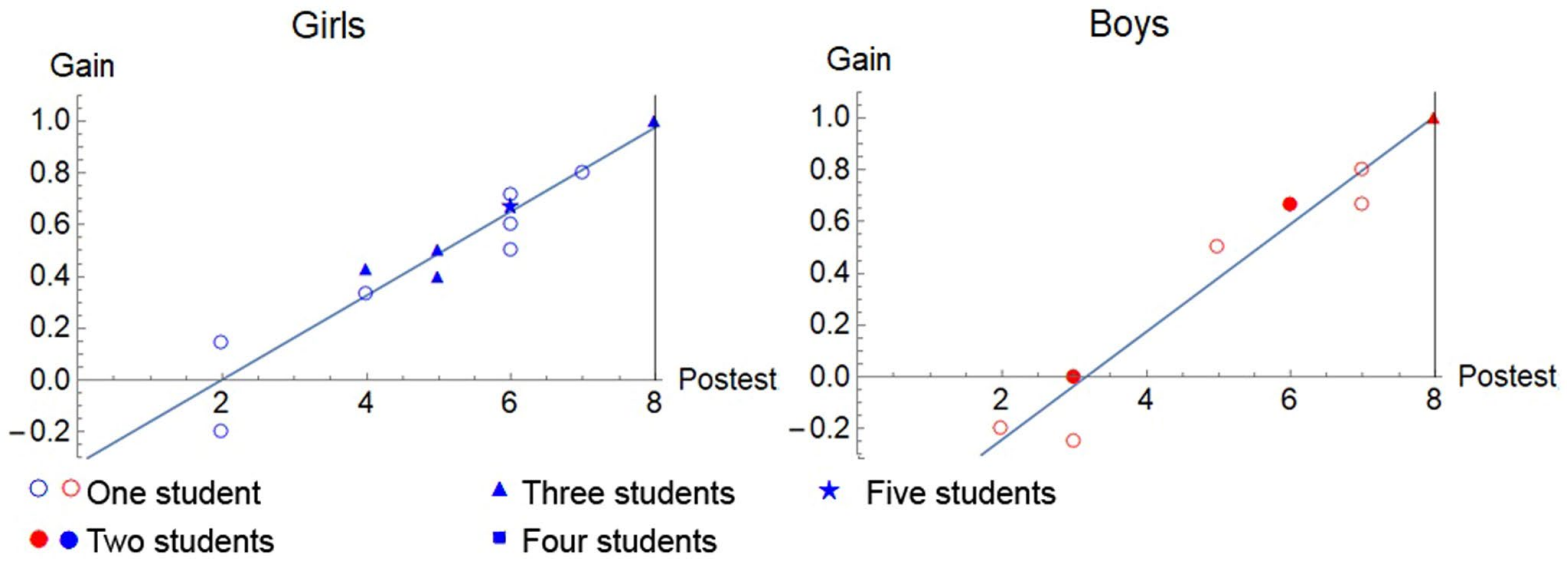
Representation of the gain against the post-test scores and their corresponding regression line in experimental group.

In Figure 3, the gain increases linearly with the scores obtained, higher for boys, in consonance with what is initially observed in the initial higher average for boys. In this case, correlation coefficients of 0.95 for girls and 0.98 for boys.

*Objective 2: Investigate the effect of the same unit on students’ appreciation and interest regarding the proposed activities and the methodology used, as well as the problems encountered and their solutions.*

Table 7 shows the results for the appreciation questionnaire answers.

**Table 7:** Student’s answers of the experimental group to the questionnaire on appreciations and interest of the pupils’ regarding the activity and the methodology used.

Item	Total		Girls		Boys	
	*M*	SD	*M*	SD	*M*	SD
1. I think I have learned a lot	3.83	0.37	3.83	0.38	3.83	0.39
2. I found the classes interesting	3.78	0.42	3.67	0.48	4.00	0.00
3. I found the way of working to be appropriate to the subject	3.64	0.48	3.58	0.50	3.75	0.45
4. I would like to work with this methodology for more of the syllabus	3.69	0.46	3.75	0.44	3.58	0.51
5. I would like to work with this methodology for whole syllabus	3.17	0.37	3.04	0.20	3.42	0.51
6. The explanations of the worksheets were adequate	3.28	0.45	3.33	0.48	3.17	0.39
7. The cooperative work was efficient	3.50	0.50	3.38	0.49	3.75	0.45
8. I have had difficulties organizing myself	1.72	0.45	1.88	0.34	1.42	0.51
9. I have carried out the activities without being distracted	3.25	0.43	3.38	0.49	3.00	0.00

M = mean, SD = standard deviation.

As can be seen, all the scores are high (except for question 8, which was formulated in a negative way), without any significant differences between the answers from girls and boys, according to the statistical analysis carried out by using a Wilcoxon non-parametric contrast (*W* = 18, *z* = −1.3337, *p* = 0.18352, for *p* < 0.05).

In particular, the items with the highest score are: *1. I think I have learned a lot* (3.83)*, 2. I found the classes interesting* (3.78) and *4. I would like to work with this methodology for more of the syllabus* (3.69). Regarding the open questions (questions 10, 11, 12 and 13), Table 8 shows the results obtained according to the categorization made.

**Table 8:** Results of the open questions by the experimental group according to the categorization made.

Question	Categories	Total	Girls	Boys
A1.	A1.1. Technical difficulties	16.67%	16.67%	16.67%
	A1.2. Difficulties with the activities (comprehension)	38.89%	41.67%	33.33%
	A1.3. None	11.11%	8.33%	16.67%
	A1.4. Organizational in terms of fulfilling the roles	44.44%	41.67%	50.00%
	A1.5. Time	22.22%	25.00%	16.67%
A2.	A2.1. They were not overcome	5.56%	8.33%	0%
	A2.2. They were overcome with help of teammates	50.00%	50.00%	50.00%
	A2.3. They were overcome by dialog	11.11%	33.33%	50.00%
	A2.4. Internet search	11.11%	8.33%	16.67%
	A2.5. Asking the teacher	27.78%	16.67%	50.00%
A3.	A3.1. Do not know	11.11%	8.33%	16.67%
	A3.2. I will be able to apply it in my daily life.	44.44%	33.33%	50.00%
	A3.3. I will be able to apply it in my academic life	66.67%	66.67%	66.67%
A4.	A4.1. The activity has been very appropriate and I have enjoyed it a lot.	72.22%	75.00%	66.67%
	A4.2. Team organisation could be better	5.56%	8.33%	0.00%
	A4.3. Include more feedback sessions	16.67%	8.33%	16.67%

Difficulties can be appreciated in carrying out the proposal, particularly organizational ones regarding fulfilling roles (44.4%) and understanding and undertaking the activities (38.89%). However, most of these difficulties were overcome with the help of other pupils (50%) or by asking the teacher (27.78%). The difficulties arising from conflicts due to sharing and performing the tasks were mainly overcome by group dialog (11.11%) and a small number were not resolved (5.56%). At the same time, a large percentage of the group considers that what they learned could be used in everyday life (44.44%) or in their academic lives (66.67%). Regarding the final questions that asked for suggestions and reflections (A4), the answers linked to liking and enjoying the activity (72.22%) stand out and were almost ten points higher among the girls (75.00%) than among the boys (66.67%). Here are some examples of student answers to this question:

*A very interesting way to learn and to work in a group; I have liked it a lot, and it has been very interesting, although some reports have taken me a long time; I have liked this way of working because we do not just have to learn theory at school. It’s also good to learn by doing things; I have liked this type of project work, but some of the activities were long and some reports were difficult to do; I think these activities have been good and not very difficult either; This activity seemed very useful to me, and we should work like this more often; It has been a good way to organize ourselves in a cooperative way, but I think it is the most suitable for this subject; The work has been very good, the photocopies are very good for studying too.*

## 6 Discussion

This proposal aims to address the scarcity of scientific literature on the incorporation of soccer into STEM education. This scarcity had already been detected in general with respect to sports in science education (Hammrich et al., 2003). Different educational programs focused on sport, such as *Science in Sports* or *Science Through Sports*, have been developed. These programs use sport as a real-world context to guide the passion it generates in high school students toward STEM disciplines, with results that indicate an improvement in attitudes toward science and scientific identity, as well as the development of skills such as critical thinking and collaborative problem solving. In particular, with an improvement in girls’ academic performance and in their attitudes toward science (Ali et al., 2021; Galoyan et al., 2022; Hammrich et al., 2003). In the proposal presented, which can be implemented in the classroom with a few sessions, cooperative learning is developed by adapting activities and roles to the exclusive context of football, where students participate sequentially in all roles (Supplementary material 2).

In the light of the results obtained, it should be noted that the initial hypothesis has been refuted: the integration of Mathematics and Physics in cooperative problem-solving with a contextualized STEM approach produces an increase in performance among pupils, particularly among girls.

As can be seen in the results, given the average scores obtained in the pre-test and the post-test, the pupils’ academic performance increased significantly after the educational intervention. In agreement with other studies undertaken (Madsen et al., 2013), the girls had initial scores that were significantly lower compared to the boys. However, after the intervention, they obtained performance levels that were equivalent to those of boys. This result agrees with other interventions in which science learning is developed by means of problem-solving, carried out using a diversity of activities with the support of technological tools, or using STEAM (*Science, Technology, Engineering, Art and Mathematics*) approaches. In those studies, the pupils worked cooperatively to solve problems using Technology and Art (Labudde et al., 2000; Queiruga-Dios et al., 2021a, 2021b; Tveita, 1999). If the contextualisation of the problems in a real-world setting is added to those factors, student performance is greater. At the same time, considering the number of questions answered correctly, a large percentage of pupils improved their academic performance (86%), with this percentage being greater among the girls (95.8%) compared to the boys (66.7%). Many of the classroom experiences incorporating Physics and Mathematics activities and that require interactive student participation have gains distributed fairly evenly distributed between 0.36 and 0.68. This gain distribution may be due to differences between the methods or differences in how the activities are implemented (Hoellwarth and Moelter, 2011). Furthermore, as indicated, the average gain in the United States populations for courses with traditional learning methodology was 0.23 and for courses with interactive learning methodology, it was 0.48 (Hake, 1998). That gain value is exceeded in the approach given here by the boys (0.47) and, to a greater extent, by the girls (0.54). The gains obtained in other studies in which there was a change of approach in Physics teaching were also exceeded (Finkenberg and Trefzger, 2019). In this case, comparing traditional teaching (0.37) and a flipped classroom approach, gain values were obtained of 0.37 and 0.46, respectively.

In the international PISA tests, the scores have a significant difference for girls and boys in the averages for OECD countries and for Spain, in which the girls have an average performance significantly lower in Mathematics than that for boys (OECD, 2019). Although the evolution of the results over the last decade has produced a narrowing, the gap persists, and, furthermore, the proportion of boys with the highest levels (5 and 6) of the Mathematics scores is greater. In Spain, 8% of boys reach that level in Mathematics, whereas only 5.5% of girls do so. That trend is repeated in the OECD average. Regarding sciences, the gender gap in average performance for 2018 is smaller than that seen in Mathematics, with girls beating the boys by a significant difference in the OECD average, which is not the case in Spain, although a trend downwards in the average scores for both groups is perceptible, particularly for boys. At the same time, the proportion of boys in the highest levels of science is greater than that of girls. The boys are a percentage majority in the high and low levels, whereas the girls are clearly the majority in the intermediate levels (Ministerio de Educación y Formación Profesional (MEFP), 2020; OECD, 2019). On the other hand, in the contextualized comprehensive STEM approach used for this research, girls obtain, on average, scores similar to those for boys. Furthermore, looking at the gains, almost all the girls obtain medium to high values, whereas the gains for boys are uniformly distributed over the three ranges according to Hake’s defined categories (1998). These values are better than the results from the control group, where both girls and boys had greater percentages in the medium and high gain ranges.

Regarding pupil appreciation, their evaluation of the experience is very positive. The classes were interesting for the pupils, who feel that they have learned a lot from the syllabus and, furthermore, would like to continue working with the approach that they developed for the problem-solving activities. With the methodology used in the real context of the sport of soccer, it has been possible to introduce problem-solving activities to the pupils in an agreeable way and keep them interested in the subject (Gravemeijer et al., 2017). The pupils show a positive attitude toward learning and problem-solving in Mathematics and Physics. In this sense, it should be stressed that 75.00% of girls have made observations in this category. *The activity has been very appropriate and, I have enjoyed it a lot*. That is, the girls have declared to a greater extent a positive evaluation of the didactic approach used in the classroom. This, presumably, could be due to the contextualized STEM approach, allowing them to work on the syllabus in a way that is more suitable to their interests by giving a sense of reality to the Mathematics and Physics. Although these feelings could condition future choices made by the pupils regarding scientific subjects (Dávila-Acedo, 2017), it is important to make STEM disciplines agreeable subjects so that all pupils study them, regardless of whether they intend to continue their studies in training that is directly linked to them. In particular, it is important that it is taught in classrooms in a way that encourages girls and enhances their learning and development (Labudde et al., 2000; Mujtaba and Reiss, 2013); even more so considering that an understanding of Science and Technology is necessary not only for those whose higher educational studies depend directly on it; it is also necessary for any citizen who wants to make informed decisions on any of today’s many controversial issues (Ministerio de Educación y Formación Profesional (MEFP), 2020; OECD, 2019, 2023). Extending this to STEM disciplines, the syllabus and pedagogical approaches used in these disciplines must be analysed with a view to improving pupil performance in general, and particularly decreasing the gender gap (Clark-Blickenstaff, 2005).

At the same time, a large percentage of pupils state they had organizational difficulties in terms of fulfilling their roles. However, these difficulties have not been serious, bearing in mind the score obtained for question *7. The cooperative work was efficient* (3.50). The difficulties were overcome through dialog among peers. Thus, presumably, the context generated has an impact on the improvement of teamwork skills. It can be seen, therefore, that cooperative work, together with active methodologies such as problem-based learning, improves the efficacy of science subjects, and this performance is greater when the activity is developed in a way connected to the real-world. A key stage in this methodology is feedback, with which the teacher guides the pupils in scientific problem-solving (Queiruga-Dios et al., 2019). However, this teacher-student and teacher-team feedback also facilitates the resolution of other types of problems or conflicts that may arise between students.

As limitations in this study, the size of the sample used and its geographical limits should be indicated, so the conclusions can be considered exploratory. However, the study could serve as a basis for future research. Furthermore, while some of the results were obtained from a test to assess academic performance, other results are based on the subjective responses provided by the students, assuming that these were given honestly and sincerely. This circumstance represents a possible limitation of the study, since the self-reported nature of the data could influence the reliability of the results. In this sense, it would be appropriate to incorporate other types of observational records reported by a researcher outside the classroom, which would reduce possible bias, and the exchange of information between the teacher who is implementing the activities and the observing researcher. Furthermore, when replicating the experience and implementing learning contexts in real-world contexts, it is essential to consider the key role of the teacher in the involvement and commitment of the students. Guidance, continuous monitoring, systematic feedback and support in solving difficulties contribute significantly to strengthening students’ self-confidence and improving their performance in the proposed tasks. In this sense, those students who perceive class activities as interesting and who trust in their ability to achieve success in a given educational context tend to develop greater commitment in their affective, behavioral, cognitive and emotional dimensions (Pedler et al., 2020; Tas et al., 2019).

In future research lines, it would be interesting to study how using active methodologies in problem-solving for Physics and Mathematics in a real-world context STEM approach can influence lifelong learning, considering the gender variable. In this sense, it would also be necessary to research whether the role of teachers as influential agents in attitudes related to working habits and curiosity is also influential in creating lifelong learning habits (Bicer and Lee, 2023; Radulović et al., 2022), and to study the difference between girls and boys. Additionally, it would be interesting to analyse whether a relationship exists or not between these educational approaches and dropping out of education, also looking at how the gender variable has an influence. Another line of future research will be the evaluation of the metacognitive strategies developed by students in the study of solving problems in Physics and Mathematics in real-world contexts through Technology and Engineering.

## 7 Conclusion

This work presents a detailed description of a STEM integration experiment by means of cooperative problem-solving that gives meaning to the information used and employs digital and technological tools in an everyday context. This can help provide insight to schools and teachers who are increasingly interested in engaging students in STEM educational models (Morrison et al., 2020). Secondly, a detailed analysis was carried out on the performance and appreciation of pupils regarding the proposal for learning/teaching. It provides, therefore, practical evidence of the implementation of STEM curriculum innovations that are currently scarce (Gale et al., 2020).

Integration of Physics and Mathematics with a STEM approach results in a significant enhancement in the academic performance of girls and boys, with a notable increase observed in the case of girls and pupils with lower academic levels. The gender variable tends to be a significant predictor of pupil scientific-technological performance, with girls usually obtaining a lower average (Madsen et al., 2013; Rodríguez-Mantilla et al., 2018), although even though there is no difference between girls and boys in terms of capability (Queiruga-Dios et al., 2019). Intervening in this improvement of academic performance are several factors that are involved in the increase of interest in the disciplines of Physics and Mathematics: the problem-solving methodology, the inclusion of technologies that improve analysis, and the alternate use of cooperative work and individual activities. This can improve the self-assessment of pupils with regard to their own mathematical and scientific abilities to improve their level of self-efficacy, which will have an effect, presumably, by increasing the number of scientific vocations for both girls and boys (Redmond and Gutke, 2020; Weeden et al., 2020).

With the described teaching approach, the pupils are encouraged, especially girls, to establish connections between the disciplines of Physics and Mathematics, which makes it possible to transfer knowledge with a higher order of thought between them and also to understand how they are connected. The “real-world” context in this proposal was soccer, a sport that is often related to a male stereotype in many countries. However, the idea is that the pupils, regardless of their gender, see Mathematics and Physics as connected to each other, with Technology and Engineering, and to their setting. Without doubt, it is possible to find many contexts for classroom work in an integrated STEM approach. It is even possible to change and diversify these contexts on each occasion, adapting them to the features of the school, the pupils, and the teacher. Thus, the pupils will see everything they learn at school as connected to the real-world. This means that the idea underlying this STEM proposal can be extended to all pupils and all educational centers. Soccer was chosen as a real-world context because it is a sport with an increasingly large number of fans, and, although it is still considered a predominantly male sport and highly stereotyped, an evolution is taking place. Thus, at an international level, there is a very significant growth in the number of women who practice this sport, with numerous educational campaigns to break these stereotypes, some of them promoted by UEFA (Union of European Football Associations) and FIFA (Fédération Internationale de Football Association) (Harris, 2005; Vezzali et al., 2023; Yiapanas, 2025).

Finally, the authors want to stress the importance of rethinking the pedagogical and methodological approaches for STEM disciplines. This makes them more friendly, avoiding gender bias, increasing interest, drop-out rates and, presumably increasing scientific vocations. The importance of the role of teachers in the scientific and technological vocations of pupils must be taken into account. The influence that teachers can wield should be nurtured, as this can have an effect on the decisions pupils take regarding the paths they choose in their learning from a very early age (Barone and Assirelli, 2020; Weeden et al., 2020). Teachers must guide the teaching-learning process and support pupil self-efficacy (Morrison et al., 2020), which is a strong predictor of achievements and persistence in the field of STEM disciplines (Redmond and Gutke, 2020).

## Data Availability

The raw data supporting the conclusions of this article will be made available by the authors, without undue reservation.

## References

[ref1] Ali R. Bhadra J. Siby N. Ahmad Z. Al-Thani N. J. (2021). A STEM model to engage students in sustainable science education through sports: a case study in Qatar. Sustain. For. 13:3483. doi: 10.3390/su13063483

[ref2] Ausubel DP Novak JD Hanesian H (1976/1983). Psicología educativa: Un punto de vista cognoscitivo [educational psychology: A cognitive point of view]. México: Trillas.ṣ

[ref3] Barone C. Assirelli G. (2020). Gender segregation in higher education: an empirical test of seven explanations. High. Educ. 79, 55–78. doi: 10.1007/s10734-019-00396-2

[ref4] Barone C. Schizzerotto A. Abbiati G. Argentin G. (2017). Information barriers, social inequality, and plans for higher education: evidence from a field experiment. Eur. Sociol. Rev. 33, jcw050–jcw096. doi: 10.1093/esr/jcw050 29193014

[ref5] Becker K. Park K. (2011). Effects of integrative approaches among science, technology, engineering, and mathematics (STEM) subjects on students’ learning: a preliminary meta-analysis. Int. J. STEM Educ. 12, 23–37.

[ref6] Beichner R. J. (1994). Testing student interpretation of kinematics graphs. Am. J. Phys. 62, 750–762. doi: 10.1119/1.17449

[ref7] Bicer A. Lee Y. (2023). Effect of STEM PBL embedded informal learning on student interest in STEM majors and careers. J. Math. Educ. 12, 57–73. doi: 10.26711/007577152790038

[ref8] Bokova I. G. (2018). Cracking the code: Girls’ and women’s education in science, technology, engineering and mathematics (STEM). France: UNESCO.

[ref9] Bybee R. (2010). Advancing STEM education: a 2020 vision. Tech. Eng. Teach. 70, 30–35.

[ref10] Chang D. F. Chang Tzeng H. C. (2018). Patterns of gender parity in the humanities and STEM programs: the trajectory under the expanded higher education system. Stud. High. Educ. 45, 1108–1120. doi: 10.1080/03075079.2018.1550479, PMID: 39918549

[ref11] Clark-Blickenstaff J. (2005). Women and science careers: leaky pipeline or gender filter? Gend. Educ. 17, 369–386. doi: 10.1080/09540250500145072

[ref12] Cohen J. A. (1992). Power primer. Psychol. Bull. 112, 155–159. doi: 10.1037/0033-2909.112.1.155, PMID: 19565683

[ref13] Dare E. A. Keratithamkul K. Hiwatig B. M. Li F. (2021). Beyond content: the role of STEM disciplines, real-world problems, 21st century skills, and STEM careers within science teachers’ conceptions of integrated STEM education. Educ. Sci. 11:737. doi: 10.3390/educsci11110737

[ref14] Dávila-Acedo M. A. (2017). Las emociones y sus causas en el aprendizaje de Física y Química, en el alumnado de Educación Secundaria [emotions and their causes in the learning of physics and chemistry in secondary education students]. Eur. Secur. 14, 570–586. doi: 10.25267/Rev_Eureka_ensen_divulg_cienc.2017.v14.i3.05

[ref15] de Souza R. R. Toebe M. Mello A. C. Bittencourt K. C. (2023). Sample size and Shapiro-Wilk test: an analysis for soybean grain yield. Eur. J. Agron. 142:126666. doi: 10.1016/j.eja.2022.126666

[ref16] Ding L. Chabay R. Sherwood B. (2013). How do students in an innovative principle-based mechanics course understand energy concepts? J. Res. Sci. Teach. 50, 722–747. doi: 10.1002/tea.21097

[ref17] Eccles J. (2011). Gendered educational and occupational choices: applying the Eccles et al. model of achievement-related choices. Int. J. Behav. Dev. 35, 195–201. doi: 10.1177/0165025411398185, PMID: 39917414

[ref19] Finkenberg F. Trefzger T. (2019). Flipped classroom in secondary school physics education. J. Physics 1286:012015. doi: 10.1088/1742-6596/1286/1/012015

[ref20] Gale J. Alemdar M. Lingle J. Newton S. (2020). Exploring critical components of an integrated STEM curriculum: an application of the innovation implementation framework. Int. J. STEM Educ. 7:5. doi: 10.1186/s40594-020-0204-1

[ref21] Galoyan T. Barany A. Donaldson J. P. Ward N. Hammrich P. (2022). Connecting science, design thinking, and computational thinking through sports. Int. J. Instr. 15, 601–618. doi: 10.29333/iji.2022.15134a

[ref22] Gravemeijer K. Stephan M. Julie C. Lin F. L. Ohtani M. (2017). What mathematics education may prepare students for the society of the future? Int. J. Sci. Math. Educ. 15, 105–123. doi: 10.1007/s10763-017-9814-6

[ref23] Hake R. R. (1998). Interactive-engagement versus traditional methods: a six-thousand-student survey of mechanics test data for introductory physics courses. Am. J. Phys. 66, 64–74. doi: 10.1119/1.18809

[ref24] Halloun I. Hake R. Mosca E. Hestenes D. (1995). Force concept inventory. Available at: https://www.physport.org/assessments/assessment.cfm?A=FCI (Accessed February 4, 2025)

[ref25] Hammrich P. L. Fadigan K. Richardson G. M. Livingston B. (2003). Sisters in sport science: a sport-oriented science and mathematics enrichment program. Elec. J. Res. Sci. Math. Educ. 7:3.

[ref26] Harris J. (2005). The image problem in women’s football. J. Sport Soc. 29, 184–197. doi: 10.1177/0193723504273120

[ref27] Hochberg K. Kuhn J. Müller A. (2018). Using smartphones as experimental tools—effects on interest, curiosity, and learning in physics education. J. Sci. Educ. Technol. 27, 385–403. doi: 10.1007/s10956-018-9731-7

[ref28] Hoellwarth C. Moelter M. J. (2011). The implications of a robust curriculum in introductory mechanics. Am. J. Phys. 79, 540–545. doi: 10.1119/1.3557069

[ref29] Johnson D. W. Johnson R. T. (1999). Making cooperative learning work. Theory Pract. 38, 67–73. doi: 10.1080/00405849909543834

[ref30] Jonassen D. Strobel J. Gottdenker J. (2005). Model building for conceptual change. Interact. Learn. Environ. 13, 15–37. doi: 10.1080/10494820500173292

[ref31] King D. Ritchie S. M. (2012). “Learning science through real-world contexts” in Second international handbook of science education (Dordrecht: Springer), 69–79.

[ref32] Labudde P. Herzog W. Neuenschwander M. P. Violi E. Gerber C. (2000). Girls and physics: teaching and learning strategies tested by classroom interventions in grade 11. Int. J. Sci. Educ. 22, 143–157. doi: 10.1080/095006900289921

[ref33] Lavonen J. Byman R. Uitto A. Juuti K. Meisalo V. (2010). Students’ interest and experiences in physics and chemistry related themes: reflections based on a ROSE-survey in Finland. Themes Sci. Technol. Educ 1, 7–36.

[ref34] MacDonald A. Wise K. Tregloan K. Fountain W. Wallis L. Holmstrom N. (2019). Designing STEAM education: fostering Relationality through design-led disruption. Int. J. Art Des. Educ. 39, 227–241. doi: 10.1111/jade.12258, PMID: 39910408

[ref35] Madsen A. McKagan S. B. Sayre E. C. (2013). Gender gap on concept inventories in physics: what is consistent, what is inconsistent, and what factors influence the gap? Phys. Rev. Phys. Edu. Res. 9:020121. doi: 10.1103/PhysRevSTPER.9.020121, PMID: 39916291

[ref36] MECD (2015). Real Decreto 1105/2014, de 26 de diciembre, por el que se establece el currículo básico de la Educación Secundaria Obligatoria y del Bachillerato [Royal Decree 1105/2014, of December 26, which establishes the basic curriculum for compulsory secondary education and baccalaureate]. Spain: MECD.

[ref37] MECD (2022). Real Decreto 217/2022, de 29 de marzo, por el que se establece la ordenación y las enseñanzas mínimas de la Educación Secundaria Obligatoria [Royal Decree 217/2022, of march 29, establishing the organization and minimum teachings of compulsory secondary education]. Spain: MECD.

[ref38] Ministerio de Educación y Formación Profesional (MEFP) (2020). Panorama de la educación. Indicadores de la OCDE. Informe español [panorama of education. OECD indicators. Spanish report.]. Madrid: General Technical Secretariat.

[ref39] Morrison J. Frost J. Gotch C. McDuffie A. R. Austin B. French B. (2020). Teachers’ role in students’ learning at a project-based STEM high school: implications for teacher education. Int. J. Sci. Math. Educ. 19, 1103–1123. doi: 10.1007/s10763-020-10108-3

[ref40] Mujtaba T. Reiss M. J. (2013). Inequality in experiences of physics education: secondary school girls' and boys' perceptions of their physics education and intentions to continue with physics after the age of 16. Int. J. Sci. Educ. 35, 1824–1845. doi: 10.1080/09500693.2012.762699

[ref41] National Academy of Engineering and National Research Council (NRC) (2014). STEM integration in K-12 education: Status, prospects, and an agenda for research. Washington, DC: The National Academies Press.

[ref42] Naukkarinen J. K. Bairoh S. (2020). STEM: a help or a hinderance in attracting more girls to engineering? J. Eng. Educ. 109, 177–193. doi: 10.1002/jee.20320

[ref43] Nunnally J. C. (1967). Psychometric theory. New York: McGraw-Hill.

[ref44] O’Connor P. O’Hagan C. Myers E. S. Baisner L. Apostolov G. Topuzova I.. (2020). Mentoring and sponsorship in higher education institutions: men’s invisible advantage in STEM? High. Educ. Res. Dev. 39, 764–777. doi: 10.1080/07294360.2019.1686468

[ref45] OECD (2018). Education at a glance 2018: OECD indicators. París: OECD Publishing.

[ref46] OECD (2019). PISA 2018 results: Combined executive summaries. París: OECD Publishing.

[ref47] OECD (2023). PISA 2022 results (volume I): The state of learning and equity in education. Paris: OECD Publishing.

[ref48] Pedler M. Hudson S. Yeigh T. (2020). The teachers’ role in student engagement: a review. Aus. J. Teacher Educ. 45, 48–62. doi: 10.14221/ajte.2020v45n3.4

[ref50] Queiruga-Dios M.A. Diez-Ojeda M. Velasco-Pérez N. (2019). Utilización de las TIC en la construcción de la física: análisis de una propuesta didáctica. Comunicación presentada en La educación ante el nuevo entorno digital. Congreso Iberoamericano de docentes. Available at: http://formacionib.org/congreso-entorno-digital/0045.pdf

[ref51] Queiruga-Dios M. A. López-Iñesta E. Diez-Ojeda M. Sáiz-Manzanares M. C. Vázquez-Dorrío J. B. (2021a). Implementación de un proyecto STEAM en Educación Secundaria generando conexiones con el entorno. J. Study Educ. Dev. 44, 871–908. doi: 10.1080/02103702.2021.1925475, PMID: 39918549

[ref52] Queiruga-Dios M.Á. López-Iñesta E. Diez-Ojeda M. Vázquez-Dorrío J.B. (2021b). Technologies applied to the improvement of academic performance in the teaching-learning process in secondary students. In: Herrero Á. Cambra C. Urda D. Sedano J. Quintián H. Corchado E. (eds) The 11th international conference on EUropean transnational educational (ICEUTE 2020). ICEUTE 2020. Advances in intelligent systems and computing.

[ref53] Queiruga-Dios M.A. Velasco-Pérez N. Diez-Ojeda M. (2018). Construyendo la física a través del fútbol. Available at: https://dialnet.unirioja.es/descarga/libro/832072/2.pdf (Accessed February 4, 2025)

[ref54] Radulović B. Županec V. Stojanović M. Budić S. (2022). Gender motivational gap and contribution of different teaching approaches to female students’ motivation to learn physics. Sci. Rep. 12:18224. doi: 10.1038/s41598-022-23151-7, PMID: 36309593 PMC9617855

[ref55] Redmond P. Gutke H. (2020). STEMming the flow: supporting females in STEM. Int. J. Sci. Math. Educ. 18, 221–237. doi: 10.1007/s10763-019-09963-6

[ref56] Rennie L. Venville G. Wallace J. (2018). “Making STEM curriculum useful, relevant, and motivating for students” in STEM education in the junior secondary. eds. Jorgensen R. Larkin K. (Singapore: Springer).

[ref57] Rocard M. Csermely P. Jorde D. Lenzen D. Walwerg-Henriksson H. Hemmo V. (2007). Science education now: A renewed pedagogy for the future of Europe. Brussels: European Comission.

[ref58] Rodríguez-Mantilla J. M. Fernández-Díaz M. J. Olmeda G. J. (2018). PISA 2015: Predictores del rendimiento en Ciencias en España [PISA 2015: Predictors of science performance in Spain]. Rev. de Educ. 380, 75–96. doi: 10.4438/1988-592X-RE-2017-380-373

[ref59] Roehrig G. H. Dare E. A. Ellis J. A. Ring-Whalen E. (2021). Beyond the basics: a detailed conceptual framework of integrated STEM. Discip. Interdiscip. Sci. Educ. Res. 3, 1–18. doi: 10.1186/s43031-021-00041-y, PMID: 39928931

[ref60] Sáiz-Manzanares M. C. Queiruga-Dios M. A. García-Osorio C. I. Montero-García E. Rodríguez-Medina J. (2019). Observation of metacognitive skills in natural environments: a longitudinal study with mixed methods. Front. Psychol. 10, 1–13. doi: 10.3389/fpsyg.2019.02398, PMID: 31736820 PMC6838136

[ref61] Sanders M. (2009). STEM, STEM education, STEM mania, 68:, 20–26. Available at: https://www.teachmeteamwork.com/files/sanders.istem.ed.ttt.istem.ed.def.pdf (Accessed February 4, 2025)

[ref62] Sanders M. (2012). “Integrative STEM education as “best practice” in Explorations of best practice in technology, design, & engineering education (Vol.2). Griffith Institute for Educational Research. ed. Middleton H., 103–117.

[ref63] Shernoff D. J. Sinha S. Bressler D. M. Ginsburg L. (2017). Assessing teacher education and professional development needs for the implementation of integrated approaches to STEM education. Int. J. STEM Educ. 4, 13–16. doi: 10.1186/s40594-017-0068-1, PMID: 30631669 PMC6310389

[ref64] Stearns E. Bottia M. C. Giersch J. Mickelson R. A. Moller S. Jha N.. (2020). Do relative advantages in STEM grades explain the gender gap in selection of a STEM major in college? A multimethod answer. Am. Educ. Res. J. 57, 218–257. doi: 10.3102/0002831219853533

[ref65] Suprapto N. (2020). Do we experience misconceptions?: an ontological review of misconceptions in science. Stud. Hist. Phil. Sci. 1, 50–55. doi: 10.46627/sipose.v1i2.24

[ref66] Tas Y. Subaşı M. Yerdelen S. (2019). The role of motivation between perceived teacher support and student engagement in science class. Educ. Stud. 45, 582–592. doi: 10.1080/03055698.2018.1509778

[ref67] Thébaud S. Charles M. (2018). Segregation, stereotypes, and STEM. Soc. Sci. 7:111. doi: 10.3390/socsci7070111

[ref68] Thibaut L. Ceuppens S. De Loof H. De Meester J. Goovaerts L. Struyf A.. (2018). Integrated STEM education: a systematic review of instructional practices in secondary education. Euro. J. STEM Educ. 3:2. doi: 10.20897/ejsteme/85525, PMID: 35799004

[ref69] Thornton R. K. Sokoloff D. R. (1998). Assessing student learning of Newton’s laws: the force and motion conceptual evaluation and the evaluation of active learning laboratory and lecture curricula. Am. J. Phys. 66, 338–352. doi: 10.1119/1.18863

[ref70] Tracker (2020). Tracker video analysis and modeling tool for physics education. Available at: https://physlets.org/tracker(Accessed February 4, 2025)

[ref71] Trinidad A. Carrero V. Soriano R. M. (2006). Teoría fundamentada “Grounded Theory”. La construcción de la teoría a través del análisis interpretacional. Cuadernos metodológicos 37: 9–174. Madrid: CIS.

[ref72] Tveita J. (1999). “Can untraditional learning methods used in physics help girls to be more interested and achieve more in this subject?” in Research in science education in Europe. eds. Bandiera M. Caravita S. Torracca E. Vicentini M. (Dordrecht, Netherlands: Springer), 133–140.

[ref73] Vaarmets T. (2018). Gender, academic abilities and postsecondary educational choices. J. Appl. Res. High. Educ. 10, 380–398. doi: 10.1108/JARHE-12-2017-0155

[ref74] Vezzali L. Visintin E. P. Bisagno E. Bröker L. Cadamuro A. Crapolicchio E.. (2023). Using sport media exposure to promote gender equality: counter-stereotypical gender perceptions and the 2019 FIFA Women’s world cup. Group Process. Intergroup Relat. 26, 265–283. doi: 10.1177/13684302221075691

[ref75] Vieyra Software (2020). Physics toolbox. Available at: https://www.vieyrasoftware.net(Accessed February 4, 2025)

[ref76] Vorhölter K. Krüger A. (2021). Metacognitive strategies in modeling: comparison of the results achieved with the help of different methods. Quadrante 30, 178–197. doi: 10.48489/quadrante.23653

[ref77] Weeden K. A. Gelbgiser D. Morgan S. L. (2020). Pipeline dreams: occupational plans and gender differences in STEM major persistence and completion. Sociol. Educ. 93, 297–314. doi: 10.1177/0038040720928484

[ref78] Yiapanas G. (2025). Addressing gender inequalities in European football: key dimensions and strategies. Insight. Sports Sci. 7:711. doi: 10.18282/iss711

