# Supplementary material for: STEM approach using soccer: improving academic performance in Physics and Mathematics in a real-world context
Source: Front Psychol. 2025 Feb 24;16:1503397. doi: 10.3389/fpsyg.2025.1503397 (PMC11891190; doi:10.3389/fpsyg.2025.1503397)
Supplement: Supplementary file 1 [file Supplementary_file_1.docx]

Supplementary Material 1

# Contents of Physics and Mathematics addressed in the STEM Unit (MECD, 2015)

| **Physics** | **Mathematics** |
| --- | --- |
| Scientific research. Scalar and vector quantities. Fundamental and derived quantities. Equation of dimensions. Errors in measurement. Expression of results. Analysis of the experimental data. Information and Communication Technologies in scientific work. Research project. Necessary strategies in scientific activity. The movement. Uniform rectilinear, uniformly accelerated rectilinear, and uniform circular motions. Vector nature of forces. Forces of special interest: weight, normal, friction, centripetal. Law of universal gravitation. Pressure. Principles of hydrostatics. Kinetic and potential energies. Mechanical energy. Conservation principle. Work and power. | Planning the problem-solving process. Strategies and procedures put into practice: use of the appropriate language: (graphic, numerical, algebraic, etc.), reformulation of the problem, solve subproblems, exhaustive count, start with simple particular cases, look for regularities and laws, etc. Reflection on the results: a review of the operations used, assignment of units to the results, verification, and interpretation of the solutions in the context of the situation, search for other forms of resolution, etc. The approach of school mathematical investigations in numerical, geometric, functional, statistical, and probabilistic contexts. The practice of mathematization and modelling processes, in contexts of reality and in mathematical contexts. Confidence in one's own abilities to develop appropriate attitudes and face the difficulties of scientific work. Use of technological means in the learning process for: a) the orderly collection and organization of data. b) the elaboration and creation of graphical representations of numerical, functional, or statistical data. c) facilitate the understanding of geometric or functional properties and the performance of numerical, algebraic, or statistical calculations. d) the design of simulations and the elaboration of predictions on diverse mathematical situations. e) the preparation of reports and documents on the processes carried out and the results and conclusions obtained. f) communicate and share, in appropriate settings, information and mathematical ideas. Angle measurements in the sexagesimal system and in radians. Application of geometric knowledge to solving metric problems in the physical world: measurement of lengths, areas, and volumes. Lengths, areas, and volumes. Initiation to analytical geometry in the plane: Coordinates. Vectors. Interpretation of a phenomenon described by means of a statement, table, graph, or analytical expression. Analysis of results. |

**References**

MECD (2015) Real Decreto 1105/2014, de 26 de diciembre, por el que se establece el currículo básico de la Educación Secundaria Obligatoria y del Bachillerato [Royal Decree 1105/2014, of December 26, which establishes the basic curriculum for Compulsory Secondary Education and Baccalaureate]. MECD, Spain. Retrieved from https://www.boe.es/eli/es/rd/2014/12/26/1105
