# Supplementary material for: STEM approach using soccer: improving academic performance in Physics and Mathematics in a real-world context
Source: Front Psychol. 2025 Feb 24;16:1503397. doi: 10.3389/fpsyg.2025.1503397 (PMC11891190; doi:10.3389/fpsyg.2025.1503397)
Supplement: Supplementary file 2 [file Supplementary_file_2.docx]

Supplementary Material 2

# Class sessions

Implementation of the activities took place in eight 50-minute sessions over two weeks. To develop the activities, the pupils had a smartphone with the *PhysicsToolbox* (Vieyra Software, 2020) application installed; a PC with office programs (including a spreadsheet), Video Tracker (Tracker, 2020); a soccer ball; a tape measure; and a compressor with manometer (to inflate the ball and determine the pressure). In addition, the pupils also had other materials available that they could request from the teacher: a cord (if, for example, they wanted to build their instrument for measuring the height from a window to the ground), a bucket of water (to determine, for example, the volume of the soccer ball by submerging it), etc. Each student had to provide their own field notebook and, furthermore, each team had to write a report on each practical session and send it to the teachers.

Throughout the sessions, the pupils had to solve different types of problems, which allowed attention to be paid to diversity (Felder & Brent, 2005). The syllabus covered for the subjects of Physics/Chemistry and Mathematics for the 4^th^ grade of CSE can be found grouped in the corresponding curriculums (MECD, 2015, 2022), where the Physics contents studied by students of those ages refer practically to force and energy: a) Physics/Chemistry – Block 1. Scientific activity, Block 4. Movement and forces, Block 5. Energy; b) Mathematics: Block 1. Processes, methods, and attitudes in mathematics, Block 2. Numbers and Algebra, Block 3. Geometry, Block 4. Functions. Parts of the curriculum, logically, form part of different Blocks from previous years. The contents of the STEM unit were made and spun looking for a constructivist approach so that the students had to construct the concepts and reach conclusions based on the setting up of different experimental situations. This constructivist approach has the sense of “an approach to learning that holds that people actively construct or make their own knowledge and that reality is determined by the experiences of the learner” (Elliott et al., 2000, p. 256). Thus, the results of some of the sessions were necessary to understand and solve the situations of the following sessions. Below is a summary of the 8 sessions of the complete syllabus developed (Queiruga-Dios et al., 2018; Queiruga-Dios, 2020):

*Session 1*. The first session began with a presentation to the pupils of the proposal for cooperative work. Students were encouraged, guided, and motivated to use the basic elements of cooperation (Johnson & Johnson, 1999). For this, the following rotating roles were designed among the students, as responsible for: coordinator, secretary, recorder, and material manager. This grouping and the proposed activities favour, the development of both academic and social skills. In addition, each student was required to have their own notebook. Thus, the feedback was produced through previous and continuous instruction on the task carried out by the team and also on individual work, to increase students' interpersonal and group skills. Prior knowledge was detected (pre-test of the syllabus in mathematics and physics available in electronic supplementary materials (ESM3)). The syllabus worked on, included revision and extension of some concepts that had already been worked on in previous years: a) scalar and vector magnitudes; b) fundamental and derived magnitudes; c) motion; d) uniform rectilinear motion; e) initiation in analytical geometry of planes; coordinates; vectors. Then, general instructions for the work were given, the teams of players organized (maintaining the same groupings as for all the classes), and the “model daily report” document was provided, which gave indications on how to write the report for each activity. The students must answer questions using soccer representations as in Figure 1.a, *In the image below, a soccer player kicks the ball and scores a goal. What trajectory has the ball followed? What was the displacement?*

*Session 2*. The following points in the syllabus were worked on: a) equation of dimensions; b) errors in measurement; c) uniformly accelerated rectilinear motion; d) the vector nature of forces; e) forces of special interest: weight, friction. All this was to be done by studying the movement of a soccer ball under the action of gravity: gathering data, handling them in the spreadsheet, and representing and interpreting the space travelled/time parabola in free fall. The relativity of the motion was also studied.

With the data taken for the free fall of a soccer ball and the already known value for acceleration due to gravity (or by obtaining it from the *PhysicsToolbox* app), the pupils can represent the motion parabola and the teachers can guide them towards the meaning of its parameters. These data can be obtained, for example, from the time sequences of a motion video which also shows distance references, or by using the *Video Tracker* software (Figure 1.b).


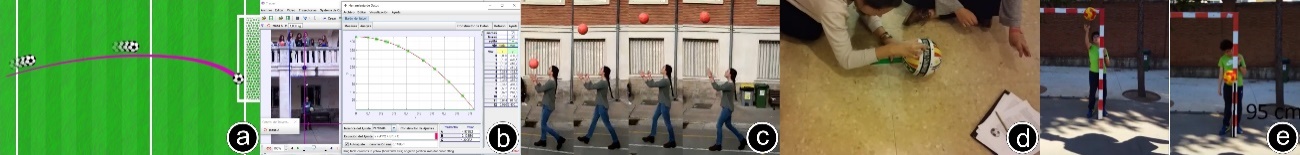


Figure 1. Several activities carried out in different sessions.

At the same time, the teachers analysed with the students and, in light of the results, the existence of errors and their causes, which is common in the study of real physical systems.

In order to study the relativity of motion, a member of the team threw the ball up vertically and caught it while moving at a constant velocity along a lateral line. Another member videoed this action while moving in parallel at the same speed. From the other lateral line, a third member, at rest, also videoed the scene. The videos were later analysed (Figure 1.c) to reach conclusions.

*Session 3*. The syllabus points dealt with in this session were: a) application of geometrical knowledge to resolve measurement problems in the physical world: measurement of lengths; b) uniform circular motion; c) measurement of angles in the sexagesimal system and in radians. These syllabus points were organized around the following activities:

- Individual problem-solving activity (with multiple solutions to resolve, taking into account geometric ideas) for homework. The pupils had to determine the velocity at which the goalkeeper must move to stop the ball regardless of the direction of a penalty shot. To do this, they had to search for information on the maximum velocity a ball can be shot and the maximum velocity that a running person can move. Finally, the pupils had to answer the question: *Why, then, is it so difficult to stop a penalty?*
- Determination of reaction speed. From the equation for uniformly accelerated motion obtained in the previous session.
- Circular motion: Determination of the maximum circumference length of the soccer ball by obtaining its volume (each team had to find a way to do this). Relationship between arc and angle, from the graphical representation of the rotated angle (radians)/space travelled (Figure 1.d). Construction of the angular velocity concept from the relationships found previously by rolling a ball across the floor and measuring the distance travelled and the time used.

*Session 4*. Teacher-pupils Feedback. Experience sharing among teams, updating and reviewing of reports.

*Session 5*. The syllabus points worked on were: a) pressure; b) principles of hydrostatics; c) kinetic and potential energy; d) mechanical energy; e) principal of energy conservation.

As homework before the session, the pupils individually revised the syllabus points regarding the kinetic theory of gases and the concept of gas pressure by using interactive materials supplied by the teachers (Newton's Apple, n.d*.*). In the classroom, the pupils were given the following questions: *How can the mass of air in the soccer ball be as certain? How can the volume of the soccer ball be calculated?*

Once some possible answers had been determined, the pupils were asked to draw the graph showing mass of air against ball pressure. The value for the air pressure inside the ball could be obtained by using the manometer on the compressor.

Finally, the pupils determined by experiment the loss of mechanical energy of the soccer ball on hitting the ground. They had to find their own references to measure heights and take videos. Figure 1.e shows some shots.

*Session 6*. The syllabus points related to work and power were worked on. For this, the pupils took part in an activity in which they jumped with their arms in distinct positions: arms in front, arms down their sides and arms towards the back. In each case, they determined the height and time of the jump by analysing the videos they took.

*Session 7*. Review of reports and feedback. The teachers generate a debate among the pupils while they analyse the reports.

*Session 8*. Evaluation: post-test survey on the syllabus, final questionnaire on appreciation.

**References**

Elliott, S. N., Kratochwill, T. R. L., and Travers, J. F. (2000). Educational psychology: Effective teaching, effective learning. *3rd* Edn. Boston: McGraw-Hill.

Felder R.M., and Brent, R. (2005) Understanding student differences. *J. Eng. Educ. 94*(1): 57-72. <https://doi.org/10.1002/j.2168-9830.2005.tb00829.x>

Johnson, D.W., and Johnson, R.T. (1999). Making cooperative learning work. *Theory. Pract. 38*(2): 67-73. <https://doi.org/10.1080/00405849909543834>

MECD (2015). Real Decreto 1105/2014, de 26 de diciembre, por el que se establece el currículo básico de la Educación Secundaria Obligatoria y del Bachillerato [Royal Decree 1105/2014, of December 26, which establishes the basic curriculum for Compulsory Secondary Education and Baccalaureate]. MECD, Spain. Retrieved from <https://www.boe.es/eli/es/rd/2014/12/26/1105>

MECD (2022). Real Decreto 217/2022, de 29 de marzo, por el que se establece la ordenación y las enseñanzas mínimas de la Educación Secundaria Obligatoria [Royal Decree 217/2022, of March 29, establishing the organization and minimum teachings of Compulsory Secondary Education]. MECD, Spain. Retrieved from <https://www.boe.es/eli/es/rd/2022/03/29/217/con>

Newton's Apple (n.d.). La teoría cinética [Kinetic theory]. Accessed September 19, 2023, from <http://www.lamanzanadenewton.com/materiales/aplicaciones/ltc/la_teoria_cinetica.html>

Queiruga-Dios, M.A. (2020). Recuperando la Física en Secundaria a través del fútbol. *Revista Española de Física*, *34*(3), 29-32.

Queiruga-Dios, M.A., Velasco-Pérez, and N., Diez-Ojeda, M. (2018). *Construyendo la física a través del fútbol*. A Coruña: Editorial Q. <https://dialnet.unirioja.es/descarga/libro/832072/2.pdf>

Tracker (2020). Tracker Video Analysis and Modeling Tool for Physics Education. Accessed November 19, 2023, from <https://physlets.org/tracker>

Vieyra Software (2020). Physics Toolbox. Accessed September 19, 2023, from <https://www.vieyrasoftware.net>
