# Supplementary material for: STEM approach using soccer: improving academic performance in Physics and Mathematics in a real-world context
Source: Front Psychol. 2025 Feb 24;16:1503397. doi: 10.3389/fpsyg.2025.1503397 (PMC11891190; doi:10.3389/fpsyg.2025.1503397)
Supplement: Supplementary file 3 [file Supplementary_file_3.docx]

Supplementary Material 3

# Questionnaire.

**1**. If I drop an inflated soccer ball and an uninflated one from the balcony at the same time:

a) The inflated one will reach the ground first.

b) The uninflated one will reach the ground first.

c) Neither of the above.

**2**. Draw a player on the pitch, running from left to right. Imagine that the player moves slower and slower. Represent on him what the velocity and acceleration vectors would look like.

**3**. I am told that a player has kicked the ball, across the field of play as in the following graph:

Which is the best interpretation of the graph?

a) The ball moves with constant acceleration that is not zero.

b) The ball is not moving.

c) The ball increases its velocity uniformly as it moves.

d) The ball moves at a constant velocity.

e) The ball moves with an acceleration that increases uniformly.


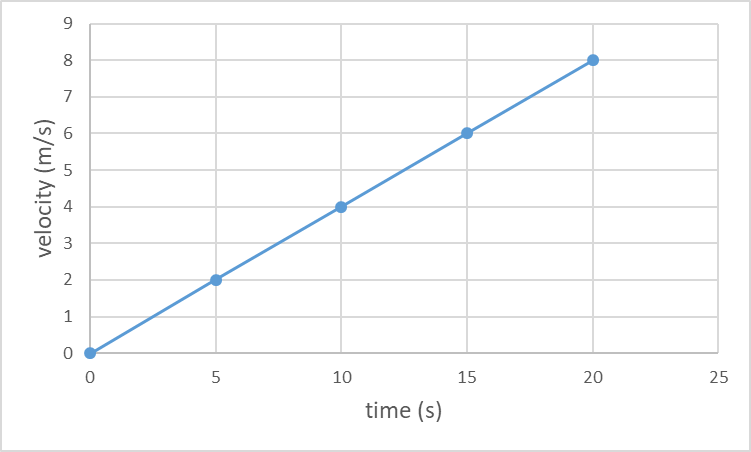
**4**. While a player is running across the soccer pitch, an observer records their velocity at specific times.

Which of the following statements best suits what is shown in the graph?

a) The player is moving over the playing field with a constant velocity.

b) The player was at rest and began to run with uniform acceleration.

c) Neither of the above is correct.

**5**. A forward passes the ball from their teammate at 60 km/h. The forward shoots at goal and the ball travels at 120 km/h. What is the kinetic energy of the ball at this moment?

a) The same, because it is the same ball.

b) Double the initial one, because it is travelling twice as fast.

c) Four times greater than initially, because the kinetic energy is proportional to the square of the velocity.

d) None of the above answers is true because these speeds cannot be reached by a soccer player.


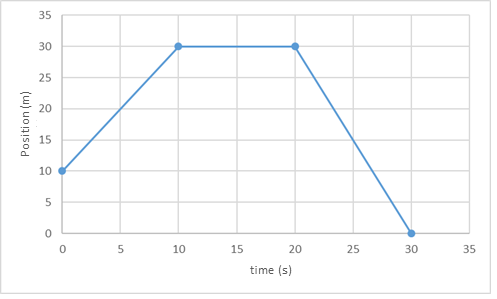
**6**. According to the graph of a player’s motion:

a) The player reaches a velocity of 30m/s

b) The player runs a total of 30m

c) In the third stretch, the player returns to the starting point more slowly.

d) The player stops for 10 s.

**7**. A soccer player and a tennis player launch their balls at the same speed. Which ball has more energy?

a) The soccer player’s ball, because it has greater mass.

b) The tennis player’s ball, because it has less mass.

c) They have the same energy at rest because they are both travelling at the same velocity.

d) They have zero energy because neither is at rest.

e) None of the above answers is correct.

**8**. A soccer ball moves according to the parabola in the figure. Which of the answers do you think could be correct?

a) The soccer ball is falling to the ground from a 35-metre-high roof.

b) It has been shot from a distance of 35 metres, and because of the friction with the grass, the ball goes increasingly more slowly.

c) A player shoots the ball with a velocity of 35 metres per second towards the goal.

d) None of the above is correct.
